# Metabolic syndrome in adults with autistic traits: associated psychological, behavioral, and biological factors in females and males – a PharmLines initiative

**DOI:** 10.3389/fpsyt.2023.1303840

**Published:** 2023-12-18

**Authors:** E. B. Warreman, L. A. Nooteboom, P. J. M. Leenen, H. M. Geurts, M. B. Terry, J. H. J. Bos, E. Hak, H. W. Hoek, E. F. C. van Rossum, R. R. J. M. Vermeiren, W. A. Ester

**Affiliations:** ^1^Department of Child and Adolescent Psychiatry, LUMC Curium, Leiden University Medical Center, Oegstgeest, Netherlands; ^2^Department of Immunology, Erasmus MC, University Medical Center Rotterdam, Rotterdam, Netherlands; ^3^Dutch Autism and ADHD Research Center, Department of Psychology, University of Amsterdam, Amsterdam, Netherlands; ^4^Dr. Leo Kannerhuis, Youz, Parnassia Group, Amsterdam, Netherlands; ^5^Department of Epidemiology, Mailman School of Public Health, Columbia University, New York, NY, United States; ^6^Groningen Research Institute of Pharmacy, PharmacoTherapy, Epidemiology and Economics, University of Groningen, Groningen, Netherlands; ^7^Department of Psychiatry, University Medical Center Groningen, Groningen, Netherlands; ^8^Parnassia Group, Youz, The Hague, Netherlands; ^9^Department of Internal Medicine, Division of Endocrinology, Erasmus MC, University Medical Center Rotterdam, Rotterdam, Netherlands; ^10^Obesity Center CGG, Erasmus MC, University Medical Center Rotterdam, Rotterdam, Netherlands; ^11^Sarr Autism Rotterdam, Youz, Parnassia Group, Rotterdam, Netherlands

**Keywords:** autism, autistic traits, cardiovascular risk, metabolic syndrome, adults

## Abstract

**Background:**

While cardiovascular diseases is highly prevalent and an important cause of mortality in autistic adults, knowledge on their increased cardiovascular risk is limited. Hence, this study aimed to investigate psychological, behavioral, and physical factors associated with metabolic syndrome (MetS) in adults with autistic traits.

**Methods:**

In total, 17,705 adults from the Lifelines Cohort were included and categorized using Autism Spectrum Quotient-10 sum-scores. The quartiles with highest (HQ-traits-group females: *n* = 2,635; males: *n* = 1803) and lowest levels of autistic traits (LQ-traits-group, *n* = idem) were analyzed. Using multivariable logistic regression, the associations between MetS and (self-reported and interviewed) psychological, behavioral, and physically measured factors in these stratified groups were investigated.

**Results:**

Among females, MetS was more common in the HQ-traits-group than in the LQ-traits-group (10.0% versus 7.5%, *p* < 0.01), while this was not the case among males (HQ-traits-group 13.8% versus LQ-traits-group 13.1%, *p* = 0.52). In both the female and male HQ-traits-group, the presence of MetS was associated with poorer self-reported health, less daily physical activity, and altered leukocyte counts.

**Conclusion:**

These findings underline the relevance of adequate cardiovascular prevention in adults with higher levels of autistic traits. Future research could gain more insight into the relationship between cardiovascular risk and autistic traits in females, and into tailored cardiovascular prevention.

## Introduction

Autism spectrum disorder (ASD) is associated with an approximate two-fold increased mortality risk (1–3). In particular, cardiovascular diseases are amongst the most common causes of death in adults with ASD (1–4). Several studies have reported an elevated risk for cardiovascular diseases in adults with ASD compared to adults without ASD, with odds ratios varying approximately from 1.3 to 2.5 (5–7). Thus, the need to reduce their cardiovascular risk is evident. Furthermore, it is relevant to investigate cardiovascular risk in the general population in order to take those adults with autistic traits, specifically females, with a late or missed ASD-diagnosis into account, by analyzing them on the presence of autistic traits, rather than only on the presence of an ASD-diagnosis (8).

Metabolic syndrome (MetS) is a globally recognized set of major cardiovascular risk factors, namely hypertension, central obesity, increased fasting glucose, and dyslipidaemia (9). The prevalence of hypertension is not higher in autistic adults than in non-autistic adults, based on a recent meta-analysis (10). To our knowledge, the prevalence of central obesity, defined by increased waist circumference, has not been studied in autistic adults or in adults with autistic traits. Regarding the prevalence of diabetes in autistic people, mixed outcomes have been reported (5, 6, 11, 12). Previous studies including autistic adults investigated different or undefined outcome measures of dyslipidaemia, resulting in contradicting results (5, 7, 11, 12). Thus, the total prevalence of MetS, defined as the presence of at least three of five criteria (9), in adults with autistic traits remains unclear.

For future development of preventive cardiovascular interventions, more insight into the psychological, behavioral, and physical factors associated with cardiovascular risk (i.e., MetS) in autistic adults is needed (7, 10). Therefore, the biopsychosocial factors that will be assessed in this study include stress, anxiety, depression, alcohol consumption, smoking, physical activity, and immunological blood markers (13–18).

We hypothesize that an increased cardiovascular risk in adults with autistic traits is associated with the degree of autistic traits and related to biopsychosocial factors. Moreover, autistic males and females have different cardiovascular risk profiles (7). Therefore, the aim of this study is to investigate the prevalence of MetS and which psychological, behavioral, and physical factors are associated with MetS in female and male adults with autistic traits.

## Methods

### Study population

Our database consisted of data from two database: the Lifelines database and the IADB.nl pharmacy database. We first included adults from the general population in the Dutch Lifelines Cohort Study. “Lifelines is a multi-disciplinary prospective population-based cohort study examining in a unique three-generation design the health and health-related behaviors of 167,729 persons living in the North of the Netherlands. It employs a broad range of investigative procedures in assessing the biomedical, sociodemographic, behavioral, biological and psychological factors which contribute to the health and disease of the general population, with a special focus on multi-morbidity and complex genetics. The Lifelines protocol was approved by the UMCG Medical ethical committee under number 2007/152” (19). We used the second assessment of the Lifelines Study, which took place between 2014 and 2017.

Next, the Lifelines data from the 37,924 participants who submitted an autism questionnaire (AUTQ) in 2019 were combined with the medication data from the University of Groningen IADB.nl pharmacy prescription database. “This is a growing database that contains prescription data for more than 20 years from 1996 to 2016 from approximately 90 community pharmacies and covers an estimated population of 900,000 patients. Registration in the database is irrespective of health care insurance and age, gender and prescription rates among the database population have been found to be representative of the Netherlands as a whole (20), and the database has been widely used for research. Each person is individually tracked throughout the database period and prescription records contain information on the date of dispensing, the quantity dispensed, the dose regimen, the number of days the prescription is valid, the prescribing physician and the Anatomical Therapeutic Chemical code (ATC code). Each patient has a unique anonymous identifier; date of birth and gender are known. Due to the high patient-pharmacy commitment in the Netherlands, the medication records for each patient are virtually complete, except for over the counter (OTC) drugs and medication dispensed during hospitalization” (21).

**Figure 1:**
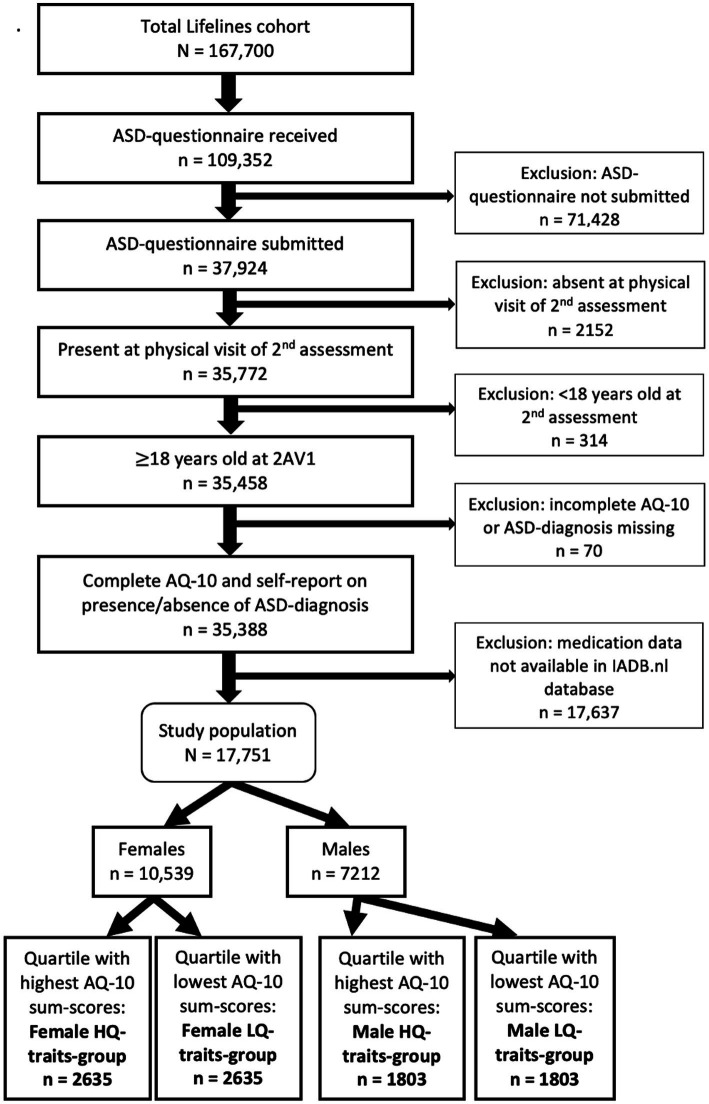
Flow diagram of study population.

For the current study (Figure 1), we included 17,705 participants, ≥18 years old at the onset of the second Lifelines assessment, who self-reported whether they had an ASD-diagnosis, and completed the short version of the Autism Spectrum Quotient (AQ-10). The 17,705 included participants were sex-stratified (10,539 females and 7,212 males) and then categorized in quartiles based on their AQ-10 sum-scores, resulting in a female quartile with highest AQ-10 sum-scores (female HQ-traits-group: *n* = 2,635), female quartile with lowest AQ-10 sum-scores (LQ-traits-group: *n* = 2,635), male quartile with highest AQ-10 sum-scores (male HQ-traits-group: *n* = 1803), and male quartile with lowest AQ-10 sum-scores (LQ-traits-group: *n* = 1803).

Of the 17,705 included participants, 198 reported having an ASD-diagnosis (1.1%). In the ASD-group (*n* = 198), 21 participants (10.6%) met the criteria for having MetS. However, G*Power analysis showed that for logistic regression using MetS as outcome and with a power of at least 0.8, in the ASD-group at least 43 participants needed to meet the criteria for MetS. Thus, the power in the diagnosed ASD-group was insufficient for performing regression.

#### Autistic community involvement

During several brainstorm sessions, our research team was advised about relevant research questions and variables by a project-group of the Dutch ‘*Academic Workplace Autism*’, which consisted of both adults with ASD and clinicians with experience treating people with ASD.

### Measures

#### Autistic traits

The AQ-10 is a valid instrument to roughly quantify the level of autistic traits in adults with average intelligence (22). It is not designed to determine the presence of an ASD-diagnosis, but it can indeed be used to investigate the degree of autistic traits in population samples (23–26). The AQ-10 consists of ten questions about the following five domains of autistic traits: attention to detail, attention switching, communication, imagination, and social skills (22). The questions are scored with a four-point Likert-scale. The minimum AQ-10 score is zero and the maximum score is 10; a higher score represents the presence of more autistic traits.

#### Metabolic syndrome

The definition of MetS was the presence of at least three of five criteria (9): an increased waist circumference (in males: 102 cm, in females: 88 cm; measured by trained Lifelines’ staff), increased fasting glucose (serum level 5.6 mmol/L and/or use of blood glucose-lowering drugs), decreased HDL-cholesterol (in males:1.0 mmol/L, in females:1.3 mmol/L, and/or use of lipid-modifying drugs), increased triglycerides (1.7 mmol/L and/or use of lipid-modifying drugs), and/or hypertension (systolic blood pressure 130 mmHg, and/or diastolic blood pressure 85 mmHg, and/or use of antihypertensive drugs). The ATC-codes used to assess the use of blood glucose-lowering drugs, lipid-modifying drugs, and antihypertensive drugs can be found in Supplementary Table S1. The use of these drugs was based on prescription in the IADB.nl database within a period of 180 days before the physical visit of the second Lifelines assessment.

#### Psychological factors

The presence of depression and anxiety were determined with a face-to-face Mini International Neuropsychiatric Interview [MINI; based on the DSM-IV-TR (27)]. Depression was defined as any current depressive disorder: major depressive disorder or dysthymia. The definition of anxiety included any current anxiety disorder: panic disorder, agoraphobia, social phobia, or generalized anxiety disorder. Long-term Difficulties Inventory (LDI) sum-scores were used to assess self-reported stress. Self-reported health was quantified with the following 5-point Likert scale RAND-question: ‘How would you rate your health generally speaking?’

#### Behavioral factors

Physical activity was determined with the following question from the Short Questionnaire to Assess Health-enhancing physical activity: “Adding everything up, on how many days per week on average are you involved in cycling, doing odd jobs, gardening, sport, or other strenuous activities for at least 30 min?” The prevalence of an average alcohol intake of at least three glasses per day [heavy drinking (28, 29)] was measured with a question from the Flower Food Frequency questionnaire (FFQ): “During the past month, how many glasses of alcoholic drinks did you drink per day on average?” Smoking was assessed with self-report regarding smoking in the past month.

#### Biological factors

Leukocyte- and subtype-counts were analyzed because they are measures of (low-grade) inflammation and a biological stress response. Chronic low-grade inflammation is an essential pathogenic factor for MetS (30, 31). Blood samples were drawn by trained Lifelines’ staff during a physical visit.

#### Covariates

Self-reported employment status and educational attainment were combined to determine socioeconomic status. Employment was defined as doing paid work for one or more hours per week. Low educational attainment included no education, primary, lower or preparatory vocational education, or lower general secondary education. Middle educational attainment was defined as: intermediate vocational education or apprenticeship, higher general secondary education, or pre-university secondary education. High educational attainment entailed higher vocational education or university. As several types psychotropic drugs can have weight gain as side effect, potentially weight-increasing antidepressants, antipsychotics, and anticonvulsants were assessed. None of the included participants used anticonvulsants. A list of the Anatomical Therapeutic Chemical (ATC) codes to identify the use of antidepressants and antipsychotics can be found in Supplementary Table S1.

### Statistical analysis

We used IBM SPSS Statistics version 25 for all data analyses. Basic characteristics, including the prevalence of MetS, were compared with univariable analyses in the following groups: female HQ-traits-group versus female LQ-traits-group and male HQ-traits-group versus male LQ-traits-group (Table 1). These univariable analyses involved Chi-square tests for categorical variables and Student’s *t-*tests or Mann–Whitney U tests for continuous variables. Next, multivariable analyses were performed in the female and male HQ- and LQ-traits-groups: psychological, behavioral, and biological factors were compared between these sex-stratified groups using multivariable regression, with correction for age and socioeconomic status (Table 2). Lastly, multivariable logistic regression with the presence of MetS as outcome measure was conducted (Table 3). These logistic regression models were executed for each of the included psychological, behavioral, and biological variables in the sex-stratified HQ-traits- and LQ-traits-groups. Age and socioeconomic status (employment and education) were included as covariates. Because of some missing data in the employment and educational attainment (see Supplementary Table S2), we performed step-by-step with three models (model 1 adjusted for age; model 2 adjusted for age and employment; model 3 adjusted for age, employment, and educational attainment). Model 3 was the most suitable as the point estimates remained similar. From the investigated potentially weight gain-inducing psychotropic drugs, only antidepressants were frequently used in our study population. Therefore, the latter logistic regression models were also performed with correction for the use of antidepressants. However, this did not result in outcomes leading to different conclusions, since the same significant outcomes were found. Transformation of skewed data was not indicated, because the assumptions of logistic regression were met based on the nature of the distributions and the large sample sizes.

**Table 1:** Basic characteristic of HQ-traits-group, LQ-traits-group, and sex-stratified subgroups.

	Females			Males		
	HQ-traits-group *n* = 2,635	LQ-traits-group *n* = 2,635	Value of *p*^a^	HQ-traits-group *n* = 1803	LQ-traits-group *n* = 1803	Value of *p*^a^
Age (mean, SD)	49.1 (12.8)	48.6 (11.7)	N.S.	51.7 (12.9)	51.9 (11.7)	N.S.
AQ-10^b^ sum score (median, IQR)	4 (4–5)	0 (0–1)	<0.01	5 (5–6)	1 (0–1)	<0.01
Ethnicity (*N*, %)						
Eastern or Western European	2,421 (91.9)	2,484 (94.3)	N.S.	1,656 (91.8)	1,677 (93.0)	N.S.
Mediterranean or Arabic	<10 (<0.4)	<10 (<0.4)		<10 (<0.6)	<10 (<0.6)	
Black	<10 (<0.4)	<10 (<0.4)		<10 (<0.6)	<10 (<0.6)	
Asian	<10 (<0.4)	<10 (<0.4)		<10 (<0.6)	<10 (<0.6)	
Other	28 (1.1)	15 (0.6)		11 (0.6)	<10 (<0.6)	
Educational attainment (*N*, %)						
Low	581 (22.0)	302 (11.5)	<0.01	389 (21.6)	186 (10.3)	<0.01
Middle	863 (32.8)	713 (27.1)	<0.01	517 (28.7)	412 (22.9)	<0.01
High	733 (27.8)	1,171 (44.4)	<0.01	546 (30.3)	860 (47.7)	<0.01
Employment (*N*, %)	1,665 (63.2)	2006 (76.1)	<0.01	1,201 (66.7)	1,348 (74.8)	<0.01
Use of antipsychotics^c^	<10 (<0.4)	<10 (<0.4)	-	<10 (<0.6)	<10 (<0.6)	-
Use of antidepressants^d^	63 (2.4)	27 (1.0)	<0.01	21 (1.2)	<10 (<0.6)	-
**Metabolic syndrome^e^ (*N*, %)**	264 (10.0)	197 (7.5)	<0.01	248 (13.8)	236 (13.1)	N.S.
WC ≥ threshold (*N*, %)	1,135 (43.1)	1,005 (38.1)	<0.01	463 (25.7)	417 (23.1)	N.S.
Hypertension (*N*, %)	960 (36.4)	839 (31.8)	<0.01	954 (52.9)	972 (53.9)	N.S.
Triglycerides ≥ threshold (*N*, %)	273 (10.4)	209 (7.9)	<0.01	425 (23.6)	421 (23.3)	N.S.
HDL-cholesterol < threshold (*N*, %)	386 (14.6)	309 (11.7)	<0.01	196 (10.9)	163 (9.0)	N.S.
Use of lipid-modifying drugs (*N*, %)	35 (1.3)	26 (1.0)	N.S.	41 (2.3)	54 (3.0)	N.S.
Increased fasting glucose (*N*, %)	<10 (<0.4)	<10 (<0.4)	N.S.	10 (0.6)	13 (0.7)	N.S.

^a^Unadjusted value of *p*s: Chi-square tests for categorical variables and Student’s *t*-tests or Mann–Whitney U tests for continuous variables. ^b^AQ-10 = short version of the Autism Spectrum Quotient. ^c^Only antipsychotics which are likely to have weight gain as side effect were included (corresponding ATC-codes: see Supplementary Table S1). ^d^Only antidepressants which are likely to have weight gain as side effect were (corresponding ATC-codes: see Supplementary Table S1). ^e^Metabolic syndrome was defined as the presence of three or more of the following criteria: (1) waist circumference (WC) above threshold: ≥88 cm in females and ≥102 cm in males, (2) hypertension: systolic blood pressure ≥130 mmHg, diastolic blood pressure ≥85 mmHg, and/or use of antihypertensive drugs, (3) triglycerides ≥1.7 mmol/L and/or use of lipid-modifying drugs, (4) HDL-cholesterol <1.3 mmol/L in females and < 1.0 in males, and/or use of lipid-modifying. Drugs, (5) fasting serum glucose ≥5.6 mmol/L and/or use of blood glucose-lowering drugs.

**Table 2:** Psychological, behavioral and biological factors: HQ-traits-group versus LQ-traits-group.

	Females				Males			
	HQ-traits-group, *n* = 2,635	LQ-traits-group, *n* = 2,635	Value of *p*^a^	Adjusted OR (95% CI)^b^	HQ-traits-group, *n* = 1803	LQ-traits-group, *n* = 1803	Value of *p*^a^	Adjusted OR (95% CI)^b^
**Psychological**								
Stress (median, IQR)	2 (1–4)	2 (1–3)	<0.01	1.17 (1.14–1.21)	2 (0–3)	1 (0–3)	<0.01	1.17 (1.13–1.22)
Self-reported health (median, IQR)	3.0 (3.0–4.0)	3.0 (3.0–4.0)	<0.01	0.64 (0.59–0.70)	3.0 (3.0–4.0)	4.0 (3.0–4.0)	<0.01	0.65 (0.59–0.71)
Anxiety disorder (*N*, %)	331 (12.6)	127 (4.8)	<0.01	2.80 (2.23–3.52)	146 (8.1)	41 (2.3)	<0.01	3.48 (2.39–5.05)
Depressive disorder (*N*, %)	190 (7.2)	55 (2.1)	<0.01	3.39 (2.42–4.74)	80 (4.4)	27 (1.5)	<0.01	2.85 (1.77–4.59)
**Behavioral**								
Alcohol use, >2 glasses/day (*N*, %)	259 (9.8)	275 (10.4)	N.S.	0.98 (0.80–1.21)	412 (22.9)	502 (27.8)	0.02	0.70 (0.58–0.84)
Physical activity, days/week (median, IQR)	4.5 (3.0–6.0)	5.0 (3.0–6.0)	<0.01	0.94 (0.91–0.98)	4.0 (2.5–6.0)	4.5 (3.0–6.0)	<0.01	0.95 (0.91–0.99)
Smoking (*N*, %)	308 (11.7)	255 (9.7)	0.02	1.14 (0.93–1.39)	212 (11.8)	250 (13.9)	N.S.	0.73 (0.58–0.91)
**Biological**								
Total leukocytes (10^E^9/L) (median, IQR)	5.80 (4.90–6.90)	5.70 (4.90–6.80)	<0.01	1.04 (1.00–1.08)	5.80 (4.90–6.83)	5.80 (5.00–6.90)	N.S.	0.99 (0.95–1.03)
Neutrophils (10^E^9/L) (median, IQR)	3.11 (2.48–3.92)	3.03 (2.43–3.78)	0.01	1.05 (0.99–1.10)	3.03 (2.49–3.76)	3.05 (2.50–3.77)	N.S.	1.00 (0.93–1.06)
Lymphocytes (10^E^9/L) (median, IQR)	1.92 (1.58–2.33)	1.91 (1.55–2.32)	N.S.	1.00 (0.91–1.11)	1.89 (1.55–2.27)	1.90 (1.54–2.28)	N.S.	0.89 (0.78–1.02)
Monocytes (10^E^9/L) (median, IQR)	0.46 (0.38–0.55)	0.45 (0.37–0.54)	<0.01	1.94 (1.24–3.04)	0.52 (0.43–0.62)	0.51 (0.42–0.62)	N.S.	0.93 (0.57–1.51)
Eosinophils (10^E^9/L) (median, IQR)	0.15 (0.10–0.23)	0.15 (0.10–0.23)	N.S.	1.01 (0.62–1.65)	0.17 (0.11–0.26)	0.18 (0.12–0.27)	N.S.	1.13 (0.65–1.96)
Neutrophil-to-lymphocyte ratio (median, IQR)	1.62 (1.26–2.12)	1.60 (1.21–2.05)	N.S.	1.06 (0.98–1.14)	1.64 (1.25–2.14)	1.63 (1.27–2.08)	N.S.	1.04 (0.95–1.14)

^a^Unadjusted *p*-values: Chi-square tests for categorical variables and Student’s *t-*tests or Mann–Whitney U tests for continuous variables. ^b^Adjusted for age and socioeconomic status (employment and educational attainment).

**Table 3:** Multivariable logistic regression with the presence of metabolic syndrome as outcome.

	Females		Males	
	HQ-traits-group, *n* = 2,635	LQ-traits-group, *n* = 2,635	HQ-traits-group, *n* = 1803	LQ-traits-group, *n* = 1803
	Metabolic syndrome (OR, 95% CI)^a^	Metabolic syndrome (OR, 95% CI)^a^	Metabolic syndrome (OR, 95% CI)^a^	Metabolic syndrome (OR, 95% CI)^a^
**Psychological**				
Stress	1.07 (1.01–1.13)	1.05 (0.97–1.13)	1.01 (0.94–1.08)	1.12 (1.03–1.22)
Self-reported health	0.53 (0.43–0.66)	0.54 (0.44–0.68)	0.59 (0.48–0.72)	0.47 (0.38–0.58)
Anxiety disorder	1.13 (0.74–1.72)	1.68 (0.91–3.10)	1.44 (0.88–2.38)	1.42 (0.58–3.50)
Depressive disorder	1.65 (1.03–2.63)	1.93 (0.79–4.69)	1.58 (0.85–2.91)	1.06 (0.31–3.65)
**Behavioral**				
Alcohol use of >2 glasses/day	1.00 (0.57–1.78)	1.87 (1.12–3.12)	1.28 (0.85–1.95)	1.84 (1.25–2.70)
Physical activity (days/week)	0.88 (0.91–0.95)	0.90 (0.83–0.98)	0.84 (0.78–0.92)	0.85 (0.78–0.92)
Smoking	1.53 (1.01–2.30)	1.51 (0.93–2.45)	1.05 (0.66–1.66)	1.69 (1.12–2.53)
**Biological**				
Total leukocytes (10^E^9/L)	1.41 (1.30–1.52)	1.42 (1.29–1.55)	1.31 (1.21–1.43)	1.20 (1.09–1.31)
Neutrophils (10^E^9/L)	1.49 (1.34–1.65)	1.56 (1.38–1.77)	1.39 (1.24–1.57)	1.45 (1.28–1.65)
Lymphocytes (10^E^9/L)	2.32 (1.87–2.87)	1.64 (1.29–2.09)	2.00 (1.54–2.59)	1.47 (1.15–1.87)
Monocytes (10^E^9/L)	6.76 (2.81–16.28)	7.11 (2.35–21.50)	13.83 (5.39–35.49)	9.50 (3.71–24.35)
Eosinophils (10^E^9/L)	2.40 (0.84–6.87)	2.94 (0.98–8.89)	1.84 (0.69–4.86)	2.28 (0.71–7.33)
Neutrophil-to-lymphocyte ratio	1.07 (0.90–1.26)	1.25 (1.04–1.49)	1.12 (0.94–1.34)	1.17 (0.99–1.39)

^a^Adjusted for age and socioeconomic status (employment and educational attainment).

## Results

### Basic characteristics

The basic characteristics of the females and males in the HQ- and LQ-traits-groups are shown in Table 1. The mean ages were not different within the female and male groups. In both the female and male HQ-traits-groups, the socioeconomic status was lower than in the female and male LQ-traits-groups.

### Metabolic syndrome

MetS was more common in the female HQ-traits-group than in the female LQ-traits-group (10.0% vs. 7.5%, *p* < 0.01, see Table 1). In contrast, among males, the prevalence of MetS in the HQ-traits-group was not different from the LQ-traits-group (13.8% vs. 13.1%, *p* = 0.52). The prevalence of MetS was higher in the male HQ-traits-group than in the female HQ-traits-group (13.8% vs. 10.0%, *p* < 0.01).

### Psychological, behavioral and biological factors associated with MetS

The psychological, behavioral, and biological factors in the female and male HQ- and LQ-traits-groups can be found in Table 2.

Table 3 shows the associations between these psychological, behavioral, and biological factors and the presence of MetS. In the female HQ-traits-group, the presence of MetS was associated with higher stress levels, poorer self-reported health, and the presence of a depressive disorder (OR 1.07, 95% CI 1.01–1.13; OR 0.53, 95% CI 0.43–0.66; OR 1.65, 95% CI 1.03–2.63; see Table 3). To explain, for example, a one-point higher score on the LDI stress questionnaire increases the odds of having MetS 1.07 times. Regarding behavioral factors, the presence of MetS was associated with less physical activity and smoking in the female HQ-traits-group (OR 0.88, 95% CI 0.91–0.95; OR 1.53, 95% CI 1.01–2.30). In other words, one more day of at least 30 min of physical activity per week decreases the odds of having MetS 0.88 times. In addition, higher total leukocyte-, neutrophil-, lymphocyte-, and monocyte-counts were associated with MetS in the female HQ-traits-group. However, in the female HQ-traits-group, the presence of anxiety disorders, alcohol use of more than two glasses per day, eosinophil-counts, and the neutrophil-to-lymphocyte ratio were not associated with the presence of MetS.

In the male HQ-traits-group (see Table 3), the presence of MetS was associated with poorer self-reported health, less physical activity, and higher total leukocyte-, neutrophil-, lymphocyte-, and monocyte-counts (OR 0.59, 95% CI 0.48–0.72; OR 0.84, 95% CI 0.78–0.92; OR 1.31, 95% CI 1.21–1.43; OR 1.39, 95% CI 1.24–1.57; OR 2.00, 95% CI 1.54–2.59; OR 13.83, 95% CI 5.39–35.49). In this male HQ-group, MetS was not associated with stress levels, the presence of anxiety or depressive disorders, alcohol use, smoking, eosinophil-counts, and the neutrophil-to-lymphocyte ratio.

## Discussion

Our study showed that in the general population, MetS is more common in females with higher levels of autistic traits than in females with lower levels of autistic traits. When comparing males with higher and lower levels of autistic traits, their prevalence of MetS was not different. These findings are concordant with a previous sex-stratified study including adults with an ASD-diagnosis (7).

With respect to the investigated psychological factors, in both females and males with higher levels of autistic traits, the presence of MetS was strongly associated with poorer self-reported health. Also, stress levels and the presence of anxiety disorders were moderately associated with MetS in females with higher levels of autistic traits. To our knowledge, these findings cannot directly be compared to other studies, since the relation between these psychological variables and MetS in adults with autistic traits has not been examined previously. It does seem that autistic traits, self-reported health, stress and anxiety disorders are interrelated, based on previous research (32–34).

Regarding the assessed behavioral factors, the presence MetS was strongly associated with less physical activity in both females and males with higher levels of autistic traits. Moreover, females and males with higher levels of autistic traits were less physically active than females and males with lower levels of autistic traits. In previous studies, adults either with an ASD-diagnosis or autistic traits also reported less physical activity (35, 36). Smoking was moderately associated with MetS in the females with higher levels of autistic traits from our study. However, in our study, females with higher levels of autistic traits did not smoke more than females with lower levels of autistic traits, which is in line with previous research in autistic adults (37). Together, especially enhancement of physical activity should be taken into account in the prevention of cardiovascular risk for adults with autistic traits.

From the investigated biological factors, MetS was strongly associated with leukocyte and several -subtype counts in both males and females with higher levels of autistic traits. This association could be explained by increased chronic stress levels in adults with higher levels of autistic traits, as psychological stress can alter these immunological variables through the hypothalamic–pituitary–adrenal axis (18). Altered immune responses due to chronic stress are interrelated with metabolic activity and increased risk for cardiovascular diseases (31, 38, 39). However, MetS itself is also related to low-grade systemic inflammation, since the total leukocyte and -subtype counts were also associated with MetS in males and females with lower levels of autistic traits.

### Strengths and limitations

The large sample size is the main strength of this study, reporting on a wide range of biopsychosocial variables in adults from a general population cohort. Furthermore, our analyses based on the participants’ level of autistic traits is a first step to better understand the increased risk for cardiovascular diseases in autistic adults and to identify cardiovascular risk profiles associated with higher level of autistic traits. Another strength of this study is the use of physically measured variables (e.g., blood pressure, fasting glucose, waist circumference, cholesterol levels) and linked medication data from the IADB.nl database to define the presence of MetS in participants.

Temporality was not examined in our study, because of the cross-sectional design. Also, the AQ-10 scores were assessed on a later moment in time (on average 4 years later) than the measures of MetS and psychological, behavioral, and biological factors. However, it has previously been investigated that the AQ-10 test–retest reliability was adequate with a time interval of 6 to 12 months (40). It could be debated whether differences in AQ-10 scores between males and females had an effect on the found associations. However, the statistical AQ-10 variance was smaller in males than in females from the HQ-traits-groups. Also, the adult AQ-10 was validated for both men and women (22). Moreover, categorization of our study population in reversed order (first into HQ-/LQ-traits-groups and then sex-categorization) did not lead to other main study results. Next, it should be noted that in the Lifelines Cohort, only people with the ability to fill in self-report questionnaires were eligible for inclusion. Thus, our study results cannot be generalized to adults with (cognitive) disabilities impacting self-report. Lastly, since 25 (12.6%) of the participants with ASD from the 198 participants with ASD in the total study population were not included in the final analysis of female and male HQ- and LQ-traits-groups, our study was not able to cover all people diagnosed with ASD in our Lifelines Cohort sample.

### Implications

Healthcare providers, such as general practitioners and psychiatrists, should be alert to assess cardiovascular risk factors when providing care for females with autistic traits, because of their increased prevalence of MetS. This implies that a wider range of females with higher levels of autistic traits, other than only those with an ASD-diagnosis based on previous research (7), should be included in timely cardiovascular preventive interventions. Next, adults with autistic traits and their healthcare providers should be educated about the factors associated with MetS in this population. Future studies could gain more insight into the pathway through which autistic traits, biopsychosocial factors, and cardiovascular risk factors interact, especially in females.

## Conclusion

In females with higher levels of autistic traits, the prevalence of MetS is higher than in females with lower levels of autistic traits. In both males and females with higher levels of autistic traits, the presence of MetS is strongly associated with poorer self-reported health, less physical activity, and altered leukocyte and -subtype counts. Earlier and adequate cardiovascular preventive measures are indicated for adults with relatively more autistic traits. To decrease morbidity and mortality of adults with high levels of autistic traits, future research should focus on implementation of cardiovascular prevention for adults with autistic traits.

## Data availability statement

The data analyzed in this study is subject to the following licenses/restrictions: All data collected for the study, including individual (pseudonymized) participant data and a data dictionary defining each field in the set, are available via the Lifelines Research Office and Statistics Netherlands (CBS). Access to this dataset and other available data from the Lifelines cohort and CBS can be requested by scientists. Access will be granted after evaluation of an application form describing the research proposal (including a data selection) and a signed Data and Material Transfer Agreement. Data will be released in a secure environment. Requests to access these datasets should be directed to Director General of Statistics Netherlands (CBS), AanvraagMicrodata@cbs.nl; https://www.lifelines.nl/researcher/how-to-apply.

## Ethics statement

The studies involving humans were approved by University Medical Center Groningen, Medical Ethical Committee. The studies were conducted in accordance with the local legislation and institutional requirements. The participants provided their written informed consent to participate in this study.

## Author contributions

EW: Conceptualization, Formal analysis, Investigation, Methodology, Visualization, Writing – original draft, Writing – review & editing. LN: Conceptualization, Supervision, Writing – review & editing. PL: Conceptualization, Writing – review & editing. HG: Conceptualization, Writing – review & editing. MT: Conceptualization, Methodology, Supervision, Writing – review & editing. JB: Data curation, Writing – review & editing. EH: Writing – review & editing. HH: Funding acquisition, Writing – review & editing. ER: Conceptualization, Writing – review & editing. RV: Conceptualization, Supervision, Writing – review & editing. WE: Conceptualization, Methodology, Supervision, Writing – review & editing.
