# Supplementary material for: Metabolic syndrome in adults with autistic traits: associated psychological, behavioral, and biological factors in females and males – a PharmLines initiative
Source: Front Psychiatry. 2023 Dec 18;14:1303840. doi: 10.3389/fpsyt.2023.1303840 (PMC10773724; doi:10.3389/fpsyt.2023.1303840)
Supplement: Supplementary file 1 [file Data_Sheet_1.docx]

**Supplementary material**

**Table S1.** Included Anatomical Therapeutic Chemical (ATC) codes

|  | **ATC codes** |
| --- | --- |
| Antihypertensive drugs | C02, C03, C04, C07, C08, C09 |
| Lipid-modifying drugs | C10A, C10B |
| Blood glucose-lowering drugs | A10A, A10B |
| Anticonvulsant drugs | N03AF01, N03AG01, N03AX12, N03AX16 |
| Antidepressants | N06AA09, N06AB08, N06AB10, N06AA21, N06AF03, N06CA01, N06AA01, N06AB03, N06AX11, N06AB06, N06AB04, N06AA12, N06CA03, N06AA10, N06AF04, N06AA04, N06AX21, N06AA02, N06AB05, N06AA06 |
| Antipsychotics | N05AX12, N05AX13, N05AC02, N05AD01, N05AA01, N05AN, N05AB03, N05AF04, N05AH02, N05AN01, N05AH04, N05AB06, N05AB02, N05AH03, N05AX08, N05AE04 |

**Table S2.** Number of missing data in covariates and main outcomes

|  | **Females** | | **Males** | |
| --- | --- | --- | --- | --- |
|  | **HQ-traits-group, n=2635** | **LQ-traits-group, n=2635** | **HQ-traits-group, n=1803** | **LQ-traits-group, n=1803** |
| **Covariates** | N (%) | N (%) | N (%) | N (%) |
| Age | 0 (0) | 0 (0) | 0 (0) | 0 (0) |
| Educational attainment | 458 (17.4) | 449 (17.0) | 351 (19.5) | 345 (19.1) |
| Employment | 197 (7.5) | 176 (6.7) | 135 (7.5) | 135 (7.5) |
| **Main outcomes** |  | 8876 |  |  |
| Metabolic syndrome | 0 (0) | 0 (0) | 4 (0.2) | 0 (0) |
| WC ≥ threshold | 0 (0) | 0 (0) | 4 (0.2) | 0 (0) |
| Hypertension | 0 (0) | 0 (0) | 0 (0) | 0 (0) |
| Triglycerides ≥ threshold | 0 (0) | 0 (0) | 0 (0) | 0 (0) |
| HDL-cholesterol < threshold | 0 (0) | 0 (0) | 0 (0) | 0 (0) |
| Use of lipid-modifying drugs | 0 (0) | 0 (0) | 0 (0) | 0 (0) |
| Increased fasting glucose | 0 (0) | 0 (0) | 0 (0) | 0 (0) |
| Stress | 205 (7.8) | 181 (6.9) | 146 (8.1) | 141 (7.8) |
| Self-reported health | 193 (7.3) | 174 (6.6) | 137 (7.6) | 135 (7.5) |
| Anxiety disorder | 663 (25.2) | 585 (22.2) | 483 (26.8) | 477 (26.5) |
| Depressive disorder | 663 (25.2) | 585 (22.2) | 483 (26.8) | 477 (26.5) |
| Alcohol use, >2 glasses/day | 1217 (46.2) | 1137 (43.1) | 646 (35.8) | 551 (30.6) |
| Physical activity, days/week | 211 (8.0) | 183 (6.9) | 152 (8.4) | 143 (7.9) |
| Smoking | 311 (11.8) | 321 (12.2) | 255 (14.1) | 218 (12.1) |
| Total leukocytes | 121 (4.6) | 114 (4.3) | 77 (4.3) | 74 (4.1) |
| Neutrophils | 138 (5.2) | 134 (5.1) | 92 (5.1) | 87 (4.8) |
| Lymphocytes | 138 (5.2) | 134 (5.1) | 92 (5.1) | 87 (4.8) |
| Monocytes | 138 (5.2) | 134 (5.1) | 92 (5.1) | 87 (4.8) |
| Eosinophils | 157 (6.0) | 152 (5.8) | 108 (6.0) | 100 (5.6) |
| Neutrophil-to-lymphocyte ratio | 138 (5.2) | 134 (5.1) | 92 (5.1) | 87 (4.8) |
